# Left bundle branch area pacing - restoring the natural order: A Case Report

**DOI:** 10.1097/MD.0000000000021602

**Published:** 2020-08-07

**Authors:** Catalin Pestrea, Alexandra Gherghina, Florin Ortan, Gabriel Cismaru, Rosu Radu

**Affiliations:** a Department of Interventional Cardiology, Brasov County Emergency Clinical Hospital; b 5th Department of Internal Medicine, Cardiology-Rehabilitation, “Iuliu Hatieganu” University of Medicine and Pharmacy Cluj-Napoca, Romania.

**Keywords:** left bundle branch block, his pacing, pacing electrode, threshold, sensing, AV block

## Abstract

**Introduction:**

Recent studies have shown that His-bundle pacing could be an alternative in patients requiring cardiac resynchronization therapy as it is comparable or better in terms of amelioration of ventricular activation, narrowing of the QRS complex, or clinical outcomes. However, in case of high threshold at the level of His-bundle or inability to correct conduction through a diseased His-Purkinje system other option should be searched like left bundle pacing.

**Patient concerns:**

A 77-year-old man presented to the Emergency Department for dizziness and dizziness and lightheadedness due to an intermittent 2:1 atrioventricular block with a QRS complex morphology of a major left branch block.

**Diagnosis:**

Given the documented symptomatic 2:1 AV block, according to the European Guideliness the patient was considered to have a class 1 indication of permanent double chamber cardiostimulation.

**Interventions:**

A lead delivery system with a C315 His catheter and a Select Secure 3830 69 cm pacing lead were placed at the His bundle area with important narrowing of the QRS complex but with an unacceptable high threshold. The delivery system was moved towards the apex 1,5 cm and the lead screwed deep into the septum until capture of the left bundle branch was achieved with complete normalization of the conduction troubles.

**Outcomes:**

At 3 month follow-up the patient was asymptomatic and the pacing and sensing thresholds remained at same values as during implantation: 0.75/0.4 ms and 14 mV respectively.

**Conclusion:**

Left bundle-pacing represents the next step of His-Purkinje system pacing to overcome all difficulties related to His-bundle pacing.

## 1 Introduction

In patients that require cardiac pacingHis bundle pacing is a far superior option to right ventricular pacing as it demonstrated lower mortality, reduction in heart failure hospitalizations and upgrade to CRT.^[1,2]^ The 2018 AHA Guidelines included His-bundle pacing for the management of patients with bradycardia and conduction delay.^[3]^ It recommends His-bundle pacing in patients with an ejection fraction of 36 to 50% (class IIa) and to patients with AV block at the suprahisian level (class IIb). Recently studies have shown that His-bundle pacing could be an alternative in patients requiring cardiac resynchronization therapy as it is comparable or better in terms of amelioration of ventricular activation, narrowing of the QRS complex, or clinical outcomes.^[4]^ However in case of high threshold at the level of His-bundle or inability to correct conduction through a diseased His-Purkinje system other option should be searched. In our report we describe the case of a patient requiring permanent pacing in whom left bundle pacing was implemented to overcome technical difficulties related to His-bundle pacing.

## 2 Case report

A 77-year-old man presented to the Emergency Department of the Brasov County Emergency Clinical Hospital for dizziness and dizziness and lightheadedness due to an intermittent 2:1 atrioventricular block with a QRS complex morphology of a major left branch block (QRS duration = 140 ms).

The lab test showed no pathological aspects, and echocardiography revealed a non-dilated left ventricle with preserved systolic function, but important intra and interventricular dyssynchronism bue to LBBB as well as moderate mitral and tricuspid valvular regurgitation.

**Figure 1:**
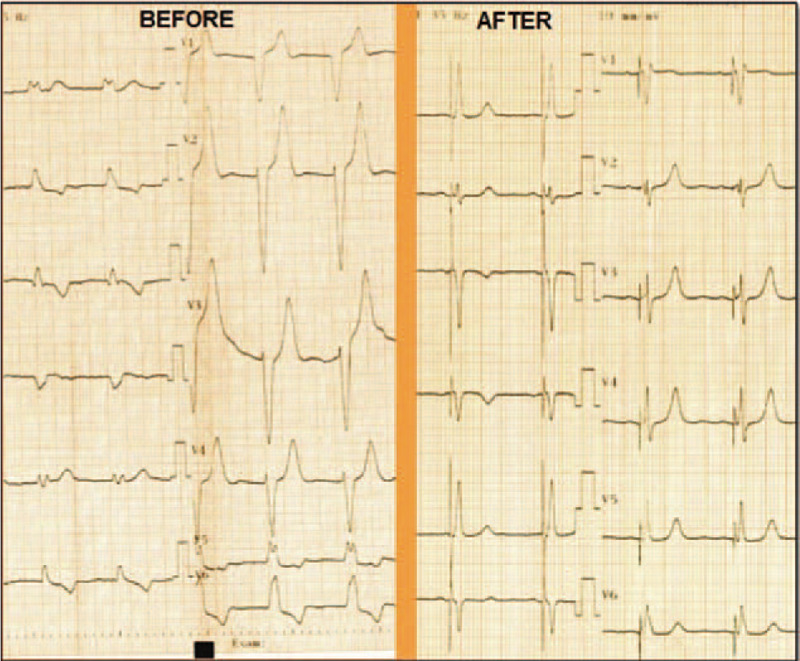
Twelve-lead electrocardiogram at baseline (BEFORE) and following left bundle branch pacing (AFTER). The baseline ECG demonstrates wide LBBB with a QRS duration of 180 ms. After left bundle branch stimulation, the QRS duration decreases to 100 ms. ECG = electrocardiograph.

**Figure 2:**
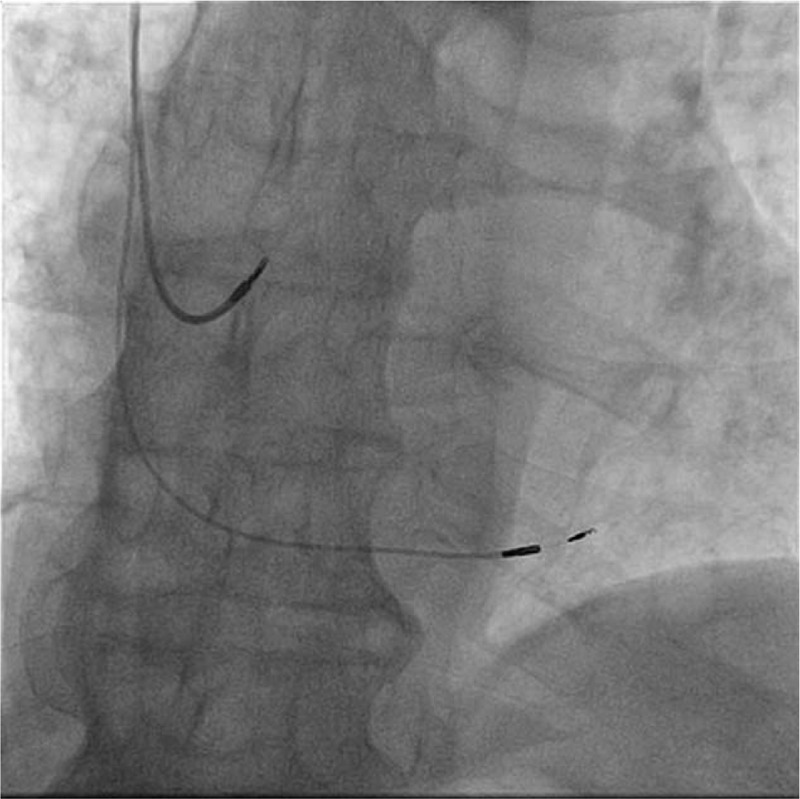
Fluoroscopic antero-posterior view of the atrial lead and deep interventricular septal lead. Compared to His bundle pacing, in left bundle branch area pacing the active lead is moved 1,5 cm more apically and screwed deep into the interventricular septum.

**Figure 3:**
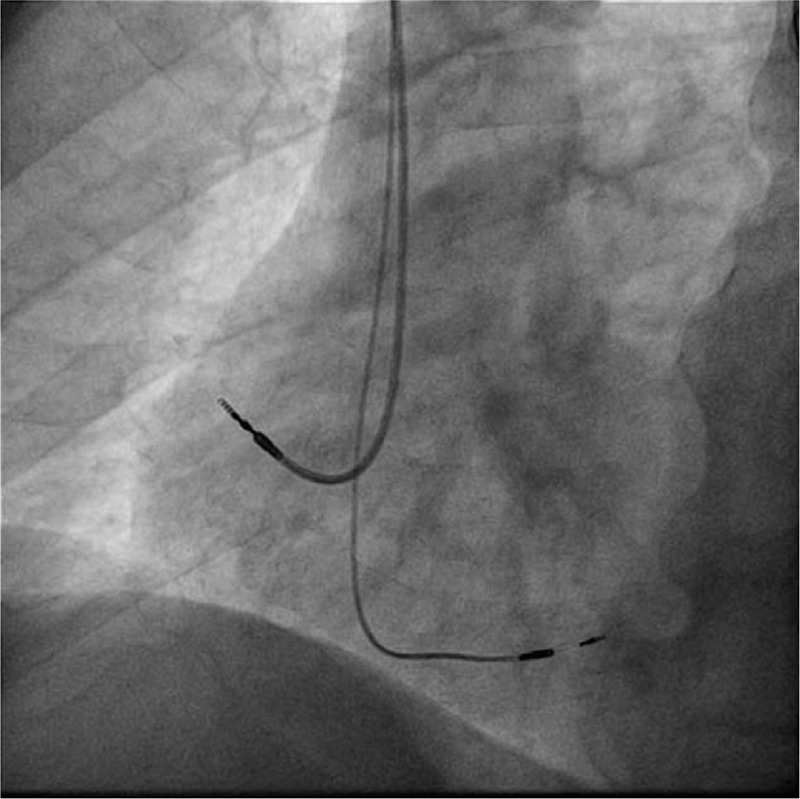
Fluoroscopic left-anterior oblique view of the atrial lead and deep interventricular septal lead. The atrial lead is towards the lateral atrial wall and the ventricular lead is oriented towards the interventricular septum.

**Figure 4:**
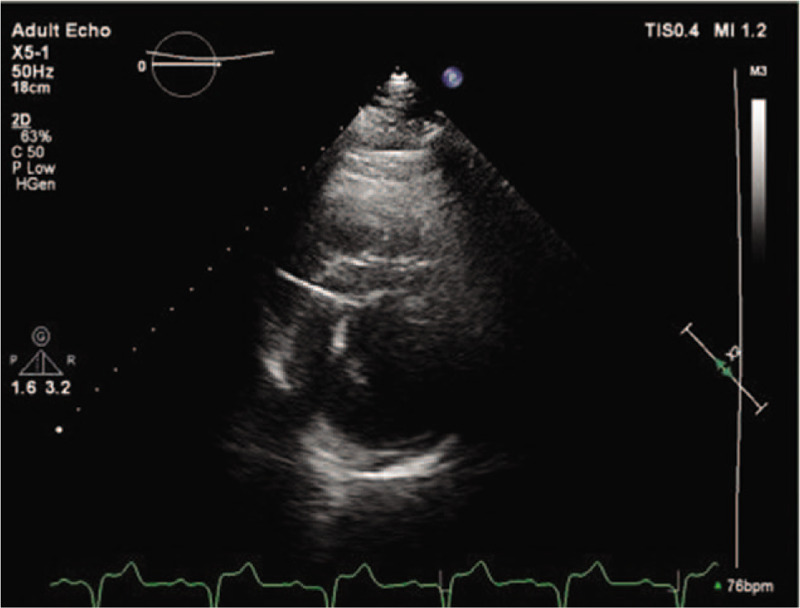
Echocardiographic parasternal short axis view shows the active fixation lead penetrating up to 10 mm into the interventricular septum, almost reaching the left ventricular endocardium.

Given the documented symptomatic conduction trouble at the level of AV node, according to the European Guideliness the patient was considered to have a class I indication for permanent dual-chamber cardiac pacing. The first attempt was to stimulate the His bundle using the technique already described in literature.^[5]^ Using a left axilary venous access and a 7-French sheath placed inside the vein, a C315 His sheath (Medtronic Minneapolis MN) was advanced over a guidewire inside the right atrium and placed near the tricuspid annulus. A SelectSecure 3830 – 69 cm pacing lead (Medtronic Minneapolis, MN) was introduced inside the sheath and mapping of the His bundle region in the unipolar mode was performed until a distal His bundle electrogram was recorded. Pacing at that level showed a significant narrowing of the QRS, but due to high pacing threshold (5 V/1ms) the position was considered unacceptable. So using the His bundle as reference in Fluoroscopy, the delivery system (sheath and lead) was moved approximately 1.5 cm towards the apex of the right ventricle and the lead was deeply screwed into the interventricular septum with continuous monitoring of the stimulated QRS complex, until the complex QRS narrowed significantly (QRS duration = 100 ms). A Qr morphology was obtained in lead V1 during unipolar stimulation, a sign that the probe was near the left ventricular endocardium and the left branch was captured distal to the block level (Fig. 1). The lead was rotated passively while monitoring the impedance and QRS duration, and the final place showed an excellent pacing threshold: 0.75 V / 0.4 ms and detection 14 mV (Figs. 2 and 3). The sheath was then removed and the lead was anchored to the pectoral muscle. Secondary, an atrial lead was placed in the right atrial appendage. Echocardiography revealed the presence of the ventricular lead tip deep into the septum just under the left endocardium (Fig. 4). Furthermore, echocardiography showed complete resolution of the inter and intraventricular dyssynchronism with an ejection fraction of 60%. At 3 months follow-up, the patient was asymptomatic and the pacing and sensing thresholds remained at the same values as during implantation: 0.75 V/0.4 ms and 14 mV, respectively

## 3 Discussion

In the last decade, the “physiologic stimulation” of the conduction system has been intensively studied and 2 techniques were implemented: His-bundle stimulation and Left bundle-stimulation. In patients with left bundle branch block the level of the block might be at the His bundle or in the proximal portion of the left branch.^[6]^ Therefore, capturing the conduction system beyond the site of block might normalize conduction of the electrical impulse through the remaining His-Purkinje system.

Huang et al. demonstrated that in patients with LBBB and nonischemic cardiomyopathy, His bundle- pacing normalizes the QRS complex in up to 80% of patients.^[7]^ However in 25% of patients with LBBB, the His bundle-pacing cannot correct the conduction disease, probably due to a more distal level of block requiring very high amplitudes, which was also the case of our patient. It has been demonstrated that pacing the left branch distal to the site of the conduction block is technically feasible and might associate a lower pacing threshold.^[8]^ In our reported case, the level of block was probably more distal, at the proximal level of the left bundle branch, therefore His-bundle pacing could not correct it with low pacing energies. However, by pacing the left bundle branch, the QRS complex become narrow with absence of intra or interventricular dyssynchronism. The 2 methods can be used as an alternative to each other: if the attempt to stimulate the His-bundle fails (inability to obtain a narrow QRS complex, increased pacing threshold, atrial or ventricular undersensing), deep septal stimulation can be used, aiming to capture the left bundle area, using the same C315 sheath and SelectSecure 3830 lead.

Many articles and systematic reviews recommend His-bundle pacing as a valuable technique. Similarly, left bundle-pacing represents the advancement of His-Purkinje system pacing to overcome all difficulties related to His-bundle pacing. Nevertheless, left bundle-pacing which is more recently introduced in clinical practice needs to be carefully investigated by future studies with emphasis on cardiac function improvement, safety and long-term performance of the pacing lead.

In summary, left bundle branch area pacing represents the next step of His-Purkinje system pacing to overcome all difficulties related to His-bundle pacing in achieving cardiac electrical resynchronization therapy

## Author contributions

**Resources:** Catalin Pestrea, Alexandra Gherghina, Florin Ortan.

**Supervision:** Catalin Pestrea, Cismaru Gabriel, Radu Rosu

**Visualization:** Catalin Pestrea, Alexandra Gherghina, Florin Ortan, Cismaru Gabriel, Radu Rosu

**Writing – original draft:** Catalin Pestrea, Cismaru Gabriel

**Writing – review & editing:** Catalin Pestrea, Cismaru Gabriel, Alexandra Gherghina, Florin Ortan, Radu Rosu.

## References

[1] Janek S Thisaranie H Beaudette D Safety and efficacy of AAIR pacing in selected pacing with sick sinus syndrome. Medicine 2018;97:e12833. 30334983 10.1097/MD.0000000000012833 PMC6211929

[2] Sharma PS Dandamudi G Naperkowski A Permanent His-bundle pacing is feasible, safe, and superior to right ventricular pacing in routine clinical practice. Heart Rhythm 2015;12:305–12. 25446158 10.1016/j.hrthm.2014.10.021

[3] Kusumoto FM Schoenfeld MH Barrett C 2018 ACC/AHA/HRS guideline on the evaluation and management of patients with bradycardia and cardiac conduction delay. A report of the American College of Cardiology/American Heart Association Task Force on Clinical Practice Guidelines and the Heart Rhythm Society. J Am Coll Cardiol 2018;1097:38984–8.

[4] Sharma PS Dandamudi G Herweg B Prmanent His bundle pacing as an alternative to biventricular pacing for cardiac resynchronization therapy: a multicenter experience. Heart Rhythm 2018;15:413–20. 29031929 10.1016/j.hrthm.2017.10.014

[5] Vijayaraman P Dandamudi G How to perform permanent His bundle pacing in routine clinical practice. Heart Rhythm 2016;13:1362–6. 27016475 10.1016/j.hrthm.2016.03.040

[6] Upadhyay GA Cherian T Shatz DY Intracardiac delineation of septal conduction in left bundle branch block patterns: mechanistic evidence of left intra-hisian block circumvented by his pacing. Circulation 2019;139:1876–88. 30704273 10.1161/CIRCULATIONAHA.118.038648

[7] Huang W Su L Wu S Long-term outcomes of His bundle pacing in patients with heart failure with left bundle branch block. Heart 2019;105:137–43. 30093543 10.1136/heartjnl-2018-313415

[8] Huang W Su L Wu S A novel pacing strategy with low and stable output: pacing the left bundle branch immediately beyond the conduction block. Can J Cardiol 33:1736e1–3. 10.1016/j.cjca.2017.09.013 29173611

